# Retinal involvement in acute thrombotic thrombocytopenic purpura: a case report

**DOI:** 10.1186/s12886-020-01719-z

**Published:** 2020-11-19

**Authors:** A. G. N. M. K. Bandara, Anne Thushara Matthias

**Affiliations:** Colombo South Teaching Hospital, Kalubowila, Dehiwala, Western Province Sri Lanka

**Keywords:** Cotton wool spots in retina, Thrombotic Thrombocytopenic Purpura, Therapeutic Plasma Exchange, Case report

## Abstract

**Background:**

Thrombotic thrombocytopenic purpura (TTP) is a life-threatening hematological condition associated with deficiency in ADAMTS13. Ocular manifestations associated with TTP are uncommon.

**Case presentation:**

Here we report a case where a 44 year old female patient with a history of symptomatic anemia and cotton wool appearance in retina during ophthalmological examination and subsequently, she was diagnosed to have TTP. The proper management with Therapeutic Plasma Exchange (TPE) and IV methylprednisolone improved the condition of the patient markedly.

**Conclusion:**

It concludes that even though the presence of cotton wool appearance in retina is a nonspecific sign it needs to be properly evaluated as there can be underlying serious illnesses as in our patient. Cotton wool spots can be an early sign of underlying retinal compromise and it should be identified early.

## Background

Thrombotic Thrombocytopenic Purpura (TTP) is a rare, life threatening hematological disease characterized by severe thrombocytopenia, microangiopathic hemolytic anemia, and organ ischemia associated with microvascular platelet rich thrombi. The brain is the most affected organ due to ischemia in TTP as 60% of patients have neurological symptoms at presentation, which range from headache to stroke and coma [1]. Ocular manifestations related to TTP are usually uncommon and underestimated due to the fact that they are mainly detectable in the preterminal stage of TTP [2]. Ocular involvement reported are serous macular detachments, choriocapillaris occlusion due to fibrin-platelet thrombi, retinal or vitreous hemorrhages, optic disc edema, neovascularization of the disc, central retinal vein occlusion, anisocoria, diplopia and homonymous hemianopia [3]. According to the published literature this was reported only in 14–20% of patients with TTP [4]. Here we report a case of TTP where the patient presents with a nonspecific headache and ocular manifestation of cotton wool spot in right fundus without any visual disturbances.

## Case presentation

A 44 -year-old previously healthy female was admitted with a history of generalized body weakness, malaise, exertional shortness of breath for one-week and episodic nonspecific headache for last one-month duration. There was no fever, urinary, bowel, respiratory symptoms or visual disturbances. Despite blood transfusion during her second pregnancy due to low Haemoglobin, her both pregnancies were uneventful. There were no suggestive features of autoimmune diseases or no bleeding manifestations. She had no significant past medical history or family history.

**Fig. 1:**
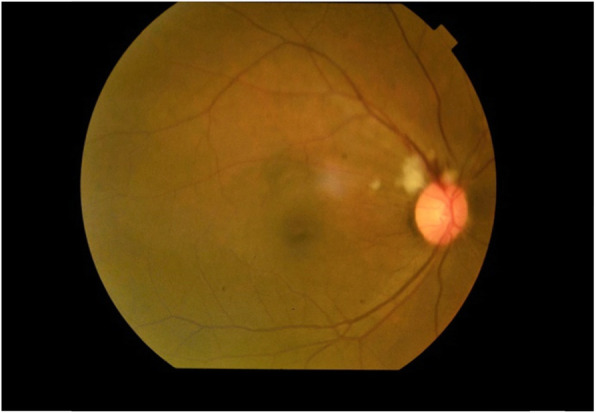
Cotton wool appearance in the Right Fundus

**Fig. 2:**
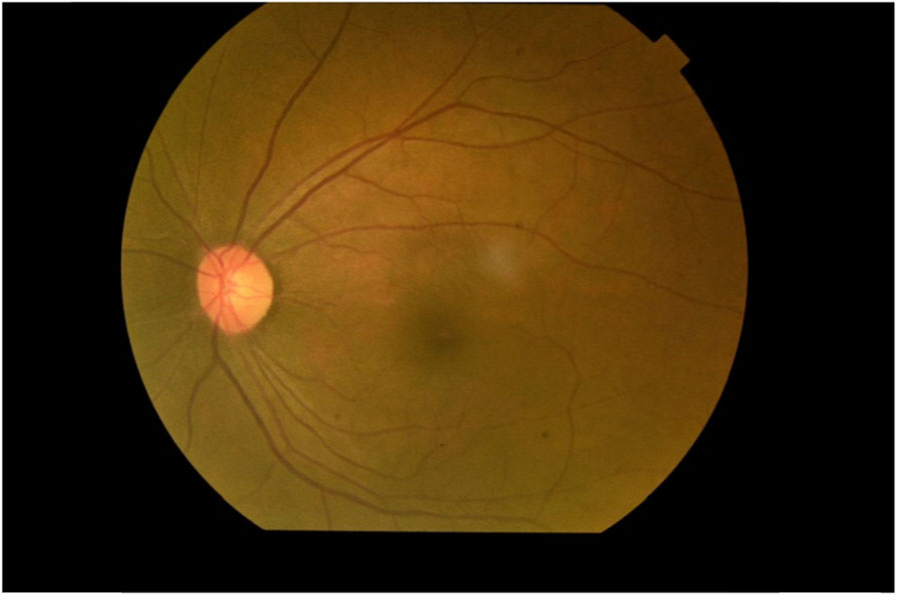
Normal Left Fundus

On hospital admission, her Glasgow Coma Scale (GCS) was 15 and she was severely pale with mild tinge of icterus. No generalize lymphadenopathy or petechial rashes. Abdominal examination revealed mild hepatomegaly without splenomegaly. Other than cotton wool appearance in the right fundus (Fig. 1), rest of the neurological examination (including left fundus (Fig. 2), visual acuity, visual field and pupillary reaction) was uneventful. Her blood pressure was 120/80 mmHg and pulse rate was 80/min. Respiratory examination was unremarkable. Random blood sugar was 123 mg/dl.

Her results of investigations showed WBC 7.84 × 10^3^/μL, Hb 6.7 mg/dl, platelet 23 × 10^3^ / μL, MCV 98.3 fL, RDW 21.7%, blood picture suggestive of Micro angiopathic hemolytic anemia, retic count 13%, DAT negative, LDH 817 U/L, serum creatinine 56.6 μmol/L, CRP < 5 mg/L, urine HCG negative, AST 34 U/L, Indirect bilirubin 29 μmol/L, INR 0.96, APTT 27 s, Fibrinogen 2.5 μmol/L, Thrombin time16.3 s, CPK 102 U/L, ESR 12, USS abdomen grade II fatty liver only, CXR and 2D Echo normal. ANA, VDRL, retroviral and other viral studies were negative.

According to the above investigation findings, she was found to have severe thrombocytopenia and micro angiopathic hemolytic anemia, which are the two key diagnosis criteria of TTP. With the other supportive investigations, the diagnosis was made as TTP.

She was managed with urgent TPE and intravenous methylprednisolone 1 g/day for 3 days initially and then converted to oral prednisolone 1 mg/kg/day. Her platelet count was markedly improved, and it was more than 150,000 after 3rd cycle of plasma exchange. She was given blood transfusion and her symptoms were improved. She was discharged with oral prednisolone and followed up at hematology clinic.

## Discussion

Cotton wool spots can be a sign of serious systemic disease. The presence of cotton wool spots in the retina is a nonspecific finding as there are many common causes such as Diabetes, Hypertension, Retinal vessel occlusion, Collagen-vascular diseases, and AIDS retinopathy [5]. The initial evaluation of cotton wool spots include blood pressure measurement, complete blood count with platelets and evaluation for diabetes. In our patient the blood pressure was normal throughout the hospital stay hence cotton wool spots are unlikely to be related to it. In this patient, there was TTP accounting for the cotton wool spots. According to the available clinical and laboratory investigations in this case, we could exclude most of the common causes of cotton wool spots in retina. This revealed the fact that the particular retinal condition was associated with TTP, which was diagnosed based on the other clinical criteria of the patient. If visual symptoms and signs are not paid attention to in evaluating the patient can have devastating consequences such as visual loss, ischemic or hemorrhagic stroke, seizures, coma or even death [6].

Ocular manifestations of TTP are rare and they depend on which blood vessels are damaged. Cotton wool spots in association with TTP to our knowledge has not been reported before. According to Percival’s clinical classification of ophthalmologic manifestations, retinal ischemia characterized by cotton wool appearance in this patient categorized as a ocular sign of systemic disorders caused by thrombotic microangiopathy [4]. However, the most common ocular manifestation in TTP is serous macular detachments [7]. Other ophthalmic manifestations include, choriocapillaris occlusion due to fibrin-platelet thrombi, retinal or vitreous hemorrhages, optic disc edema, neovascularization of the disc, central retinal vein occlusion, anisocoria, diplopia and homonymous hemianopia [3].

The onset of ophthalmological manifestations in TTP can either be early or late in the disease process. Ong et al. identified that retinal conditions and visual disturbances can be early signs of underlying systemic microangiopathy while Lambert et al. identified them to occur later in the disease process [8, 9].

Depending on the affected organ symptoms of TTP can be vary. It may include acute vision loss due to retinal pathology, blurring of vison due to papilledema or diplopia if cranial nerves are affected [10]. With the disease progression, there will be suggestive ocular symptoms of systemic damage including hypertensive retinopathy, palpebral purpura or swelling, chemosis, subconjunctival bleeding, scleral jaundice and finally retinal bleeding [4]. Therefore possibility of TTP should be considered in patients who present with vaso-occlusive disease of the retina. Further research revealed that fundus findings may be a prognostic factor in TTP [11].

Fever, thrombocytopenia, microangiopathic hemolytic anemia, neurological symptoms, and renal insufficiency were the pentad used to define TTP historically. However, this became obsolete as only less than 10% of patients with an acute TTP were presented with these five symptoms. Currently severe thrombocytopenia (typically < 30 × 10^9^/L) and microangiopathic hemolytic anemia are identified as most constant and common signs of acute TTP [1]. Apart from these two signs, this patient presented with uncommon retinal involvement of cotton wool spots in right fundus.

Early detection and appropriate therapeutic management would reduce the mortality rate of TTP up to 10 to 20% [12, 13]. Therefore, patients need to be screened for the ocular manifestations as such conditions can be an early sign of TTP [8]. TPE with replacement of plasma remains as the core of current management of TTP [14]. The clinical and hematological parameters of this patient were markedly improved following TPE and high dose of steroids.

## Conclusions

Cotton wool spots can be a sign of serious systemic disease and warrant further evaluation in certain clinical scenarios, as in this patient, a life-threatening illness was discovered. Such retinal involvement can be occurred in the course of TTP without any significant symptoms. Complete hematologic and metabolic workup should be initiated promptly due to early treatment with TPE reduces mortality rate from this rapidly fatal condition. This case report revealed that examination of fundus and vision is of paramount clinical important because TTP can lead to fatal ocular manifestations which could be detected as an early sign of the disease. Although TTP-HUS is a rare disorder, it should be considered in patients who present with vaso-occlusive disease of the retina. The mortality rate of this condition can be very high without urgent intervention, ophthalmologists may play a key role in its timely diagnosis if patients with TTP present with ophthalmological signs and symptoms.

## Data Availability

All data gathered during this study are included in this manuscript.

## Abbreviations

AIDS: Acquired Immune Deficiency Syndrome
GCS: Glasgow Coma Scale
TTP: Thrombotic Thrombocytopenic Purpura
TPE: Therapeutic Plasma Exchange
WBC: White Cell Count
MCV: Mean Corpuscular Volume
RDW: Red Cell distribution Width
DAT: Direct Antiglobulin Test
LDH: Lactate Dehydrogenase
CRP: C Reactive Protein
HCG: Human Chorionic Gonadotropin
AST: Aspartate Aminotransferase
INR: International Normalized Ratio
APTT: Activated Partial Thromboplastin Time
CPK: Creatine Phosphokinase
ESR: Erythrocyte Sedimentation Rate
USS: Ultrasound Scan
CXR: Chest X-ray
ANA: Anti-Nuclear Antibody
VDRL: Venereal Disease research Laboratory
